# Inflammatory pseudotumour of the spleen complicated by a cholesterol granuloma: a diagnostic dilemma

**DOI:** 10.1093/jscr/rjag068

**Published:** 2026-02-17

**Authors:** Caitlin Sorour, Eshwarshanker Jeyarajan

**Affiliations:** Department of General Surgery, Cairns Hospital, 165 The Esplanade, Cairns North, QLD, 4870, Australia; Department of General Surgery, Cairns Hospital, 165 The Esplanade, Cairns North, QLD, 4870, Australia

**Keywords:** inflammatory pseudotumour, cholesterol granuloma, spleen, splenectomy

## Abstract

Inflammatory pseudotumours (IPTs) and cholesterol granulomas (CG) are rare benign lesions that often mimic malignancy on imaging, creating significant diagnostic uncertainty. We report the case of a 67-year-old man with a long-standing calcified splenic cyst who developed new abdominal symptoms secondary to extrinsic compression of the stomach by the lesion and subsequently underwent a laparoscopic splenectomy. Histopathology confirmed an IPT with xanthogranulomatous inflammation. Six months later, a surveillance positron emission tomography scan revealed a solitary fluorodeoxyglucose-positron (FDG) avid left upper quadrant lesion concerning for malignancy in the context of newly diagnosed cutaneous squamous cell carcinoma. Laparoscopic resection and subsequent histopathology demonstrated a CG at the splenectomy site. This case highlights the diagnostic challenges posed by IPT and CG, emphasizes their potential to mimic malignant disease radiologically, and reinforces the importance of histopathological confirmation to guide appropriate management.

## Introduction

Inflammatory pseudotumours (IPTs) of the spleen are a rare phenomenon that were first described in the literature by Cotelingam and Jaffe in 1984 [1]. IPTs may present in multiple organ systems, with the orbit and respiratory tract being the most common [1, 2]. They are thought to be benign growths that are often difficult to diagnose accurately, particularly within the spleen where they are frequently mistaken as a malignant process [3].

A cholesterol granuloma (CG) is a benign entity defined by a foreign-body giant cell reaction to cholesterol crystals [4, 5]. They are most commonly seen in the temporal bone and middle ear; however, case reports have found rare examples located throughout the body [6]. Similar to IPT, CGs are frequently mistaken for a malignancy and continue to pose a rare, but significant diagnostic dilemma [7].

Here, we describe a unique case of an IPT of the spleen that was surgically removed, complicated by a CG at the splenectomy site.

## Case report

**Figure 1:**
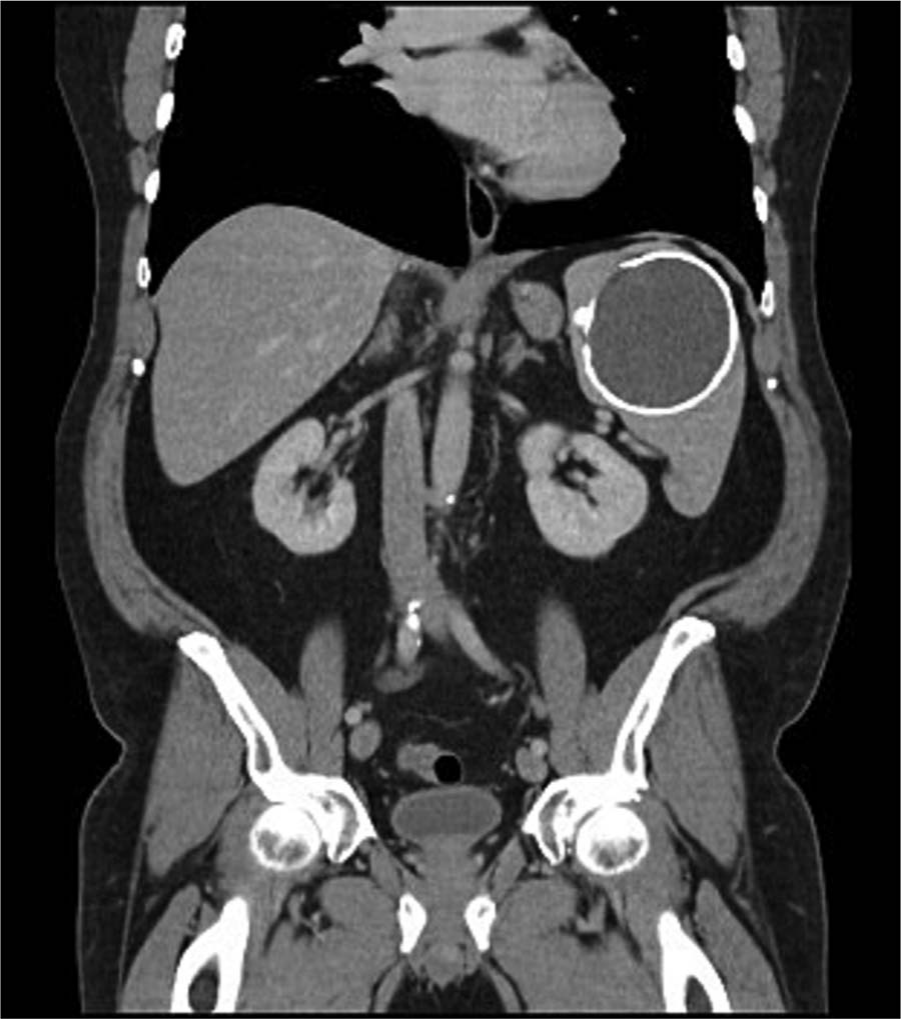
Coronal portal venous phase CT scan of the abdomen and pelvis demonstrating the calcified splenic lesion.

A 67-year-old gentleman was referred for management of a symptomatic, large splenic cystic lesion that had been found incidentally on coronary angiography in 2012. He had been worked up by his general practitioner at the time with a computed tomography (CT) scan which demonstrated a large, well-defined calcified splenic cyst measuring 8 cm in size (Fig. 1) and negative hydatid serology. On serial imaging over the following decade, the lesion was found to be mostly stable in size and was therefore only referred to a surgeon for definitive management in 2024 due to new symptoms of left upper quadrant (LUQ) abdominal pain and refractory nausea. This was accompanied by early satiety and unintentional weight loss of 10 kg over the preceding year. He had a medical history significant for coronary artery disease, dyslipidaemia, benign prostatic hyperplasia, and chronic obstructive pulmonary disease, and a surgical history significant for open epigastric, umbilical, and bilateral inguinal hernia repairs.

The suspicion was that these new symptoms were likely related to extrinsic compression of the stomach from the splenic lesion, necessitating surgical removal of the spleen; however, an intra-luminal cause was excluded with endoscopy. Endoscopy found a small hiatus hernia and reflux oesophagitis with evidence of extrinsic compression at the fundus of the stomach. Prior to proceeding with definitive surgical management, a further cardiac workup was performed which ultimately led to an angiogram necessitating percutaneous coronary intervention. As a result, the patient was placed on dual antiplatelet therapy (aspirin, clopidogrel) and the operation was delayed.

**Figure 2:**
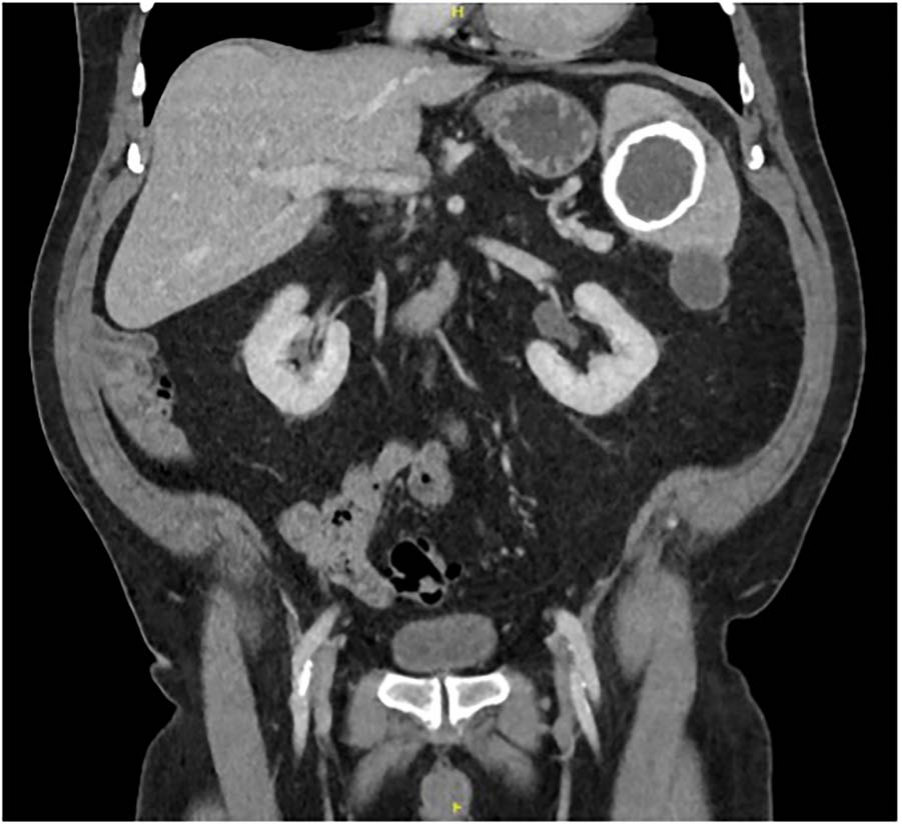
Coronal portal venous phase CT scan of the abdomen and pelvis demonstrating the subcapsular leak.

During this period of antiplatelet therapy, he experienced an episode of severe, atraumatic LUQ pain prompting presentation to the Emergency Department. A CT scan demonstrated a contained leak or haemorrhage from the large calcified splenic cyst within the subcapsular space, though no active haemorrhage was noted on arterial phase (Fig. 2). His white cell count and haemoglobin remained normal at 4.8 and 163, respectively, with no other biochemical abnormalities. He was monitored in hospital for a period before discharging once pain-free and 2 months later underwent an elective laparoscopic splenectomy. Intraoperatively, the spleen was noted to have areas of local inflammatory adhesions to the omentum and abdominal wall. Histology demonstrated an IPT like appearance with areas of necrosis and a florid xanthogranulomatous inflammatory process with focal staining for smooth muscle actin; Epstein–Barr Virus (EBV) testing was not performed. Postoperatively, his symptoms of abdominal pain, early satiety, and nausea had resolved, and an interval CT scan was scheduled in 6 months for surveillance given the atypical pathology.

**Figure 3:**
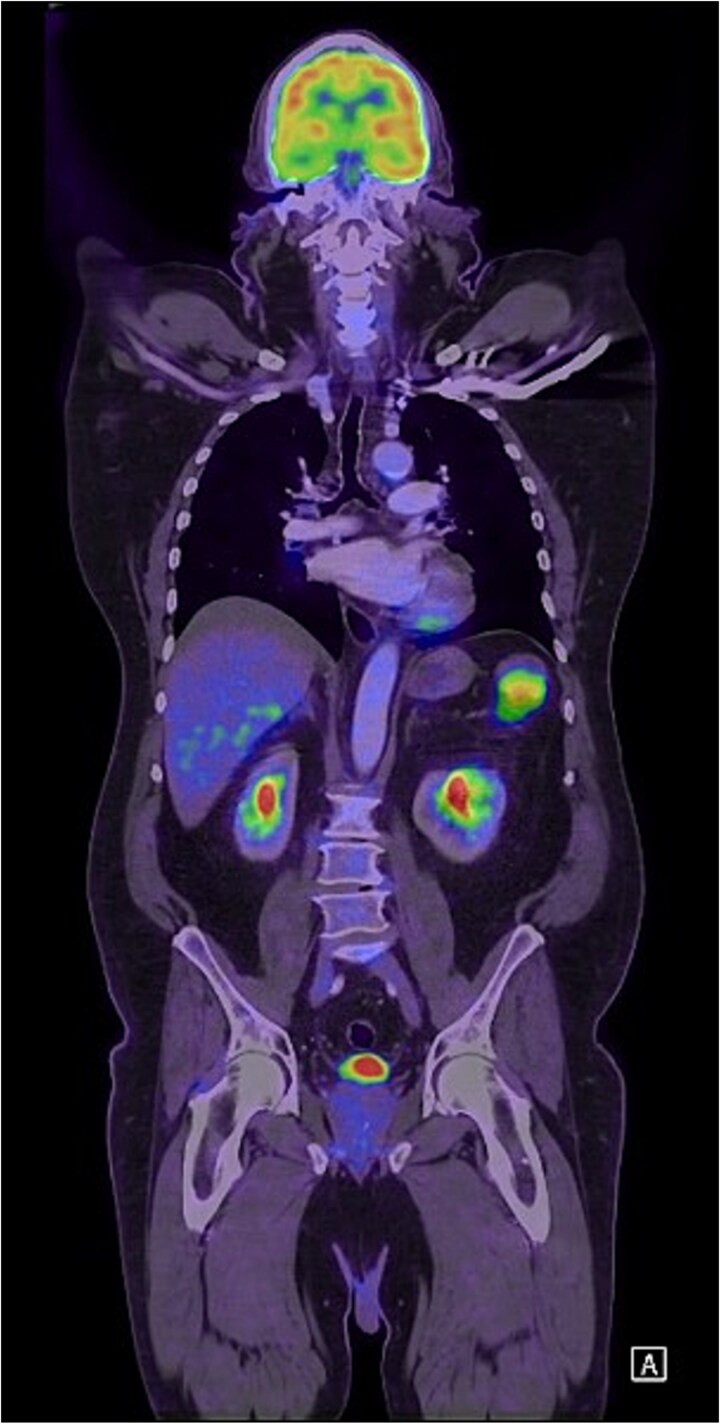
Coronal FDG PET scan of the head, neck, chest, and abdomen demonstrating a PET-avid LUQ lesion.

Unfortunately, 6 months later he was diagnosed with locally advanced squamous cell carcinoma of the face and was subsequently commenced on immunotherapy. As part of the work-up, he was re-staged with a positron emission tomography (PET) scan which found an isolated PET-avid soft tissue lesion in the LUQ measuring 51 × 37 mm in size (Fig. 3) for which he was clinically asymptomatic. Following multidisciplinary discussion, he underwent a laparoscopic soft-tissue resection with intraoperative findings of a mobile, soft tissue lesion located in the LUQ. Histology demonstrated foreign body granulomatous inflammation to cholesterol clefts in keeping with a CG. He had an uneventful postoperative recovery and remained asymptomatic on review in the surgical outpatient clinic.

## Discussion

This case highlights the diagnostic challenges of an IPT of the spleen and the rare post-splenectomy complication of a CG. IPTs of the spleen are rare benign lesions with only case series published to date [2]. The pathogenesis of splenic IPT remains debated with inflammatory, infectious, and neoplastic processes proposed and research has demonstrated a potential link with EBV [8, 9]. Splenic IPTs are often found incidentally with abdominal pain, nausea, and weight-loss being the prominent clinical symptoms of note [2, 10, 11]. Laboratory findings are often normal, and cross-sectional imaging typically reveals a discrete lesion that is difficult to distinguish from a malignancy [2, 10, 11]. Biopsy of splenic lesions carries a significant risk of haemorrhage and potential malignant seeding; therefore, upfront surgical management with a splenectomy has historically been employed [11]. This means that the diagnosis of IPT is usually made only on histological exam following splenectomy with histology demonstrating spindle-cells with myofibroblasts and an inflammatory cell component [1, 3, 9].

Similarly, CGs are rare benign lesions with only limited case-based literature published to date. The pathogenesis is thought to be due to a breakdown of blood products secondary to either inflammation or trauma, leading to the production of cholesterol crystals that causes a granulomatous reaction [6, 12, 13]. In this case, bleeding or inflammation either from the primary IPT or secondary to the splenectomy is hypothesized to be the likely aetiology. Previous case reports have found CGs to be FDG-PET avid, representing a significant diagnostic pitfall to overcome with concomitant or previous malignancy and as a result, have often required upfront surgical management to accurately differentiate using histopathology [7, 13–15].
